# Targeting the Immune system and Epigenetic Landscape of Urological Tumors

**DOI:** 10.3390/ijms21030829

**Published:** 2020-01-28

**Authors:** João Lobo, Carmen Jerónimo, Rui Henrique

**Affiliations:** 1 Department of Pathology, Portuguese Oncology Institute of Porto (IPOP), R. Dr. António Bernardino de Almeida, 4200-072 Porto, Portugal; 2 Cancer Biology and Epigenetics Group, Research Center of Portuguese Oncology Institute of Porto (GEBC CI-IPOP) and Porto Comprehensive Cancer Center (P.CCC), R. Dr. António Bernardino de Almeida, 4200-072 Porto, Portugal; carmenjeronimo@ipoporto.min-saude.pt; 3 Department of Pathology and Molecular Immunology, Institute of Biomedical Sciences Abel Salazar, University of Porto (ICBAS-UP), Rua Jorge Viterbo Ferreira 228, 4050-513 Porto, Portugal

**Keywords:** bladder cancer, epigenetics, kidney cancer, prostate cancer, targeted therapies, testicular cancer, urological cancer

## Abstract

In the last years, we have witnessed remarkable advances in targeted therapies for cancer patients. There is a growing effort to either replace or reduce the dose of unspecific, systemic (chemo)therapies, given the associated short- and long-term side effects, by introducing more specific targeted therapies as single or combination agents. Due to the well-known implications of the immune system and epigenetic landscape in modulating cancer development, both have been explored as potential targets in several malignancies, including those affecting the genitourinary tract. As the immune system function is also epigenetically regulated, there is rationale for combining both strategies. However, this is still rather underexplored, namely in urological tumors. We aim to briefly review the use of immune therapies in prostate, kidney, bladder, and testicular cancer, and further describe studies providing supporting evidence on their combination with epigenetic-based therapies.

## 1. Introduction: Focusing on Urological Cancer

Worldwide, urological cancer represents 26% and 13% of cancer incidence and mortality, respectively [1,2], and both of these statistics have been rising as a reflection of population growth and aging [3]. Disparities exist between more developed and developing countries, with new cancer events being more frequent in the former [3]. Overall, urological cancer imparts an important economic burden, which is deemed to increase, especially given the crescent influence of lifestyle factors of modern civilization including smoking and obesity [2]. Urological cancer is also very diverse; it includes the most common prostate, bladder, and kidney cancer, which are common in old-adults, and testicular cancer, less common and more prevalent in young adults and adolescents. In each of these fields, there have been improvements in patient care; however, many (and new) challenges persist, and novel biomarkers and therapies are needed, which should target the hallmarks of cancer, including the immune environment and epigenetic background [4].

In this review, we aim to briefly present some of these major challenges in the various urological cancers and how targeting the immune system might help to answer some of them. Finally, we aim to discuss how to exploit both the immune microenvironment and the epigenetic landscape in combination, to improve patient care.

## 2. Bladder Cancer

### 2.1. Major Clinical Challenges

Bladder cancer (BlCa) ranks as the second most common urological malignancy, after prostate cancer, and represents the ninth most common cancer worldwide [5]. In 2018, 549,393 new cases were diagnosed and incidence is rising. Among urological malignancies, BlCa also stands out as a particularly mortal cancer, with 199,922 deaths in 2018 [6,7]. BlCa is also a significant economic burden, representing a major challenge for healthcare systems [8]. Patients with papillary non-invasive BlCa may require frequent hospital appointments for follow-up with cystoscopy, and multiple interventions to treat recurrences, which are common in the natural history of this disease. On the other end of the spectrum, patients with invasive or even metastatic disease often require extensive surgery and systemic treatment (including immunotherapy), and management of morbidities caused by these treatments.

The most common type of BlCa is by far the one derived from the urothelial lining - the urothelial carcinoma (>90% of the cases). Upper urinary tract urothelial carcinoma is less frequent (5–10% of urothelial cancer) [9] and its biological background seems to differ from that of bladder urothelial carcinoma. In this review, we will focus mainly on urothelial carcinoma of the urinary bladder, the most studied. From a pathobiological point of view, bladder urothelial carcinoma comprises two distinct subtypes: non muscle invasive BlCa (NMIBC), the most frequent (75–80% of the cases), and muscle-invasive BlCa (MIBC), which displays an aggressive course, often culminating in metastatic disease and death [10,11]. An integrated molecular classification of BlCa has been proposed and is currently the focus of many research in the field [12,13]. Five subgroups of the disease have been identified, with specific molecular aberrations, distinct prognosis, and possible targeted teatment; this includes the luminal-infiltrated subtype (19%), with overexpression of immune-checkpoint players, being susceptible to immunotherapy [14]. 

From a clinical point of view, one third of BlCa patients present with MIBC, not amenable to treatment with transurethral resection. These might be candidates for neoadjuvant cisplatin-based chemotherapy followed by cystectomy. The overall prognosis is poor; invasive, non-metastatic BlCa has a cure rate of solely 50% [15], and metastatic disease shows a dismal prognosis. Many patients do not benefit from cytotoxic therapies, do not endure them due to important toxicities or develop resistance to cisplatin [16,17]. In this line, there is an urgent need for novel, non-invasive and cost-effective ways to follow-up patients with NMIBC during the natural history of their disease and, simultaneously, for novel treatment options for MIBC patients (combining surgery, systemic treatments, and targeted therapies) which improve patient outcome.

### 2.2. The Connection between Genetic, Epigenetic and Immune Landscape in Bladder Cancer

The immune landscape of BlCa is very rich; several immune-cell populations reside in the urinary bladder, and immunotherapies have the advantage of modulating them, namely activating favorable immune populations such as CD8+ T-lymphocytes and Th1 CD4 αβ T-lymphocytes [18]. The immune microenvironment is also linked to the molecular subtypes previously mentioned. Indeed, Ren et al. provided a immune transcriptomic analysis of BlCa genomic subtypes, showing that several pathways were differentially overactivated, like signatures related to T-cell activation, interleukin signaling and Toll-like receptor signaling [19]. This shows a link between the immune landscape and the genetic background of BlCa. The various subtypes of the disease are characterized by distinct mutations and copy-number alterations; this includes, for instance, those involving RB1 and NFE2L2, enriched in basal cancers, and those involving FGFR3 and the epigenetic enzyme KDM6A, which are prevalent in luminal subtypes). Importantly, these alterations are clinically targetable [20], and there is rationale for combining them with therapies addressing the subtype-specific immune signature of these tumors and also targeting epigenetic enzymes found to be deregulated. BlCa is among the cancers with the highest tumor mutational burden, reflecting genomic instability and tumor-specific neoantigens, which is increasingly being proposed as a biomarker for predicting the efficacy of immunotherapies [21,22,23]. Mutations in chromatin modifiers are found to be mutated in urothelial carcinoma in a frequency higher than any other cancer, which, again, argues in favor of targeted therapies against these epigenetic modifiers [24]. Overall, this stresses the need to optimally combine these agents, targeting the specific (epi)genetic landscape of the highly unstable urothelial carcinoma together with the acompanying specific immune signature, to achieve maximum efficacy.

### 2.3. Immune Therapies—Brief Overview in Bladder Cancer

Therapies based on the immune system, in the form of Bacillus-Calmette Guérin (BCG), have long been used for treating NMIBC since the 1970s, but this intravesical therapy still shows some limitations, related to side effects and treatment failure [25]. Since then, no other form of immunotherapy has been explored in BlCa until recently, with the advent of immune-checkpoint inhibitors [26]. In fact, since May 2016, the Food and Drug Administration (FDA) has approved several agents targeting the programmed death 1/programmed death-ligand 1 (PD-1/PD-L1) axis for treating platinum-refractory BlCa patients, including Nivolumab and Pembrolizumab (targeting PD-1), and also Durvalumab, Atezolizumab, and Avelumab (targeting PD-L1). Expression of PD-L1 in cancer cells results in anergy of T lymphocytes (by targeting PD-1 on their surface); this way, tumor cells escape destruction by the immune system and maintain a subverted immune-suppressive tumor microenvironment [27,28]. It has become routine, then, for pathology departments to assess PD-L1 expression by immunohistochemistry in BlCa tissue specimens [29,30]. 

Other immune-checkpoint players such as cytotoxic T lymphocyte-associated antigen (CTLA-4), which is expressed in T lymphocytes surface and binds B7 on antigen-presenting cells (APCs)—thus impeding the co-stimulatory signal necessary for T-cell activation—also constitutes a target for immune-checkpoint inhibitors, like Ipilimumab. In fact, combinations of both types of inhibitors (anti-PD-1/PD-L1 combined with anti-CTLA-4 agents) are currently undergoing clinical trials for BlCa patients [31,32], following the success observed in melanoma patients [33].

### 2.4. Role of Immunoepigenetics?

The epigenetic background of BlCa has been the focus of several research works. As an example, promoter methylation of several targets have been investigated in a liquid biopsy setting for proper monitoring of the disease, including in urine [34,35,36] (for further reading refer to [37]). Many studies have found several biomarkers informative for disease aggressiveness and patient outcome, including microRNAs, gene promoter methylation and specific histone modifications and chromatin alterations [38,39]. These epigenetic players were shown (in vitro and in vivo) to interact and regulate more or less extensively several hallmarks of cancer, including epithelial-to-mesenchymal transition (EMT), proliferation, survival, stemness, resistance to treatments, among others [40,41]. Among many examples, inhibitors of the methyltransferase EHMT2 were shown to induce apoptosis of BlCa cells [42,43,44]; natural compounds such as Honokiol (derived from *Magnolia officinalis*) inhibited BlCa growth by suppressing EZH2/miR143 [45]; and various histone deacetylase (HDAC) inhibitors were shown to be effective in reducing BlCa growth [46,47,48,49], showing synergy with other classically used therapies such as mitomycin C [50] and additionally increasing sensitivity to chemo- [51] and radiotherapy [52,53].

A summary of most recent studies addressing combination strategies between epigenetics and immune environment in BlCa is presented in Table 1 [54,55,56,57,58,59,60,61,62,63,64]. Some studies have focused on obtaining methylation-based biomarkers with predictive value, namely concerning response to BCG-therapy [55,56,57]. As previously mentioned, BCG is a commonly used therapy for NMIBC, with the main aim to impede (or at least delay) recurrence of the disease after excision (occurring eventually in >50% of patients), which might also mean shifting to invasive disease, with metastatic potential [56]. Two studies pursued a screening of several gene promoters known to be frequently involved in tumor biology, and found that hypermethylation of genes such as CDKN2B (involved in cell cycle regulation), MUS81a and MSH6 (involved in DNA repair) and THBS1 (involved in cell adhesion), associated with better response to BCG-therapy, and both studies acknowledge that the exact mechanisms for explaining these findings deserve investigation in the future [55,57]. Nevertheless, they are an example of how to bring together epigenetic phenomena and biomarkers that may predict response to immune therapies. In a similar setting, another work uncovered that demethylated PMF1 was associated with disease recurrence and poor outcome in these patients, being a biomarker of failure to respond to BCG therapy [56]. PMF1 is involved in regulating intracellular polyamine content, which in turn triggers the expression of several genes. Interestingly, it was demonstrated that high polyamine levels led to apoptosis of macrophages populating pneumocystis pneumonia [65]. This finding seems to wonderfully fit with the BCG therapy, since higher PMF1 expression mediated by demethylation of its promoter would increase polyamine levels and hence trigger apoptosis of macrophages, which would become less available to be activated by the BCG stimulus and result in treatment failure. Nonetheless, more studies are needed to investigate these processes.

Possible targets for therapeutic vaccines are the cancer testis antigens (CTAs), which have been shown to be expressed in various neoplasms, including BlCa. In a recent study, two CTAs, PRAME and CT10, were found to be expressed in 15% and 21% of bladder urothelial carcinomas, and these tumors had a poorer prognosis, with CT10-positive patients experiencing worse disease-specific survival [66]. Importantly, it has been shown that treatment with decitabine has the ability to enhance the expression of such CTAs in BlCa cell lines, making them more available to be targeted by immune therapies [54]. This strategy might be envisaged as a combination strategy for treating BlCa patients.

Epigenetic regulation of specific types of T-cells has also been explored in BlCa. Bergman et al. [58] showed that an assessment of CD4+-cell lineage commitment by looking at specific CpGs methylation status could predict the outcome of BlCa patients, with demethylation of those sites (which include FOXP3, IFNG, IL13, and IL17A) associating with lower stage and, importantly, better response to neoadjuvant chemotherapy. Moreover, Hartana et al. [59] explored the perforin gene PRF1, demonstrating that tissue-resident CD8-positive T cells show demethylation of this gene promoter, correlating with its higher expression, hence with more cytotoxic ability. Finally, Ramakrishnan et al. [62] focused on EZH2 inhibition and its effects on the immune environment. Again, a link between the important genomic landscape and epigenetic background is taken into account. The epigenetic modifiers KDM6A and SWI/SNF family are very frequently mutated in BlCa; they inhibit another epigenetic player, EZH2, a histone methyltransferase, hence loss-of-function mutations ultimately lead to EZH2 overexpression and poor prognosis. This can be explored as a therapeutic opportunity. Indeed, when exposing BlCa cells with loss-of-function mutations of KDM6A and SWI/SNF to the EZH2 inhibitor EPZ011989, this resulted in stimulation of NK cells signaling and in tumor cells death. All these strategies bring together epigenetic mechanisms regulating several subtypes of immune cells, that can be therapeutically misused to induce antitumor effects.

Non-coding RNAs are also among the epigenetic mechanisms regulating tumor progression in BlCa. Indeed, the long non-coding RNA UCA1 was found to be overexpressed in BlCa, associating with disease progression, and it is suggested that it may be used as a urine biomarker for BlCa diagnosis [67]. Moreover, its knockdown attenuated malignant features of BlCa both in vitro and in vivo [67] and, importantly, concomitant knockdown of PD-1 resulted in synergistic antitumor effect mediated by a shift in immune microenvironment, which led to increased interferon (IFN) signaling and reduced immunosuppressive pathways, as well as also enhancement of dendritic cells (DCs) maturation [60].

Recently, Segovia et al. [63] further dissected the epigenetic regulation of immune response to cancer. In this work it was shown that EHMT2 (or G9a) expression associated with poor outcome in BlCa and that targeting both EHMT2 and DNA methyltransferase (DNMT) activity (inhibitor CM-272) significantly enhances antitumor effects, that is potentiated when combining with anti-PD-L1 agent. This effect is due to immunogenic tumor death, as a consequence of increased IFN-mediated responses (histologically accompanied by infiltration of CD8-positive T cells and NK cells) and inhibition of immune-suppressive signaling, turning an immunologically “cold tumor” into a “hot tumor”.

## 3. Kidney Cancer

### 3.1. Major Clinical Challenges

Kidney cancer (KCa) is a very heterogeneous disease, with many and very distinct subtypes. Global statistics place it as the 14th most incident cancer worldwide (403,262 new estimated cases in 2018) and the 16th most deadly (175,098 cancer-related deaths in 2018) [7]. Five-year survival for patients with localized disease is good (around 90%); however, another major challenge in the field is improving outcomes of metastatic KCa patients (for which 5-year survival is much lower, around 12%) [7].

The great majority of KCa originate from the renal parenchyma – the renal cell carcinomas (RCC). There are many subtypes of RCC (recognized by the 2016 World Health Organization classification [68]), each with their own (patho)biology. The most frequent subtypes are the clear cell RCC (ccRCC), the most common and most studied (65–70% of the cases); the papillary RCC (pRCC, 15–20% of the cases); and the chromophobe RCC (chRCC, 5–10% of the cases) [68]. These entities have their own molecular background, and thus treatment approaches should also envision this, and be as much as possible subtype-specific [69,70,71,72]. An integrated analysis of each subtype has pinpointed, for instance, that ccRCC was characterized by overactivation of a specific immune signature [73].

Another challenge in KCa relates to its chemo- and radioresistance. Targeted therapies against VEGFR and the mTOR pathway have changed the paradigm of metastatic RCC [74]. However, the challenge is that patients develop resistance to these agents over time [75,76], so novel therapies are needed.

### 3.2. The Connection between Genetic, Epigenetic, and Immune Landscape in Kidney Cancer

Immune infiltration in RCC has prognostic value, with influence in patient survival [77]. Again, the immune cell populations and their prognostic implications are subtype-specific; for instance, CD8+ T-lymphocyte infiltration associated with improved overall-survival in chRCC, while in pRCC a high infiltration by M1 macrophages associated with better patient outcome. In ccRCC, increased populations of regulatory T-lymphocytes resulted in poorer survival [78]. This stresses that the heterogeneity of RCC is also extended into the immune microenvironment, in a subtype-specific way. Specific types of immune therapies, targeting the various immune cell populations, should then be reasoned based on this subtype-specificity. Even within the same histological subtype there is diversity in the microenvironment, with Chevrier et al. identifying several T-cell and tumor-associated macrophage phenotypes within ccRCC, including a specific immune signature with impact on progression-free survival [79]. ccRCC genetic background characterization pinpoints VHL as the most frequently deregulated gene, followed by mutations in several epigenetic modifiers, namely PBRM1 (43%), KDM5C (18%) and SETD2 (16%). Mutations in these genes frequently interfered with specific signaling cascades, like the cell-cycle transition or specific metabolic pathways [80]. This dependence on specific metabolic pathways and metabolic shift in RCC is further of interest for epigenetic targeted therapies, since epigenetic regulation of metabolism is described [81]. Furthermore, four types of ccRCC were identified: CD8+ inflammed, CD8- inflammed, VEGF immune desert and metabolic immune desert, characterizatized by a conjugation of specific immune signatures and genomic alterations [80], a finding that may further guide targeted treatment for these patients. Despite the relevance of genomic alterations and genomic instability, most recent data demonstrate that the immunogenicity of ccRCC is not explained by mutational load; targeting DNA/histone epigenetic modifications may help increase the efficacy of immune targeted therapies [82]. 

### 3.3. Immune Therapies – Brief Overview In Kidney Cancer

Similar to BlCa in respect to BCG, some forms of immunotherapy have been used for treating metastatic RCC patients. Indeed, before 2005, the cytokines interferon-alpha and interleukin-2 (IL-2) were the routinely used and available treatments for these patients [83], demonstrating that subverting the immune environment in KCa was an effective way of fighting this disease. Interleukin-2 (IL-2), for instance, stimulates the cytotoxic activity of T-lymphocytes against malignant cells; however, its non-specificity is responsible for severe side effects, and hence its use is decreasing [84].

Since then, and with the advent of immune-checkpoint inhibitors, several clinical trials have shown and are still addressing the effect of combining agents targeting PD-L1/PD-1 with anti-VEGFR tyrosine kinase inhibitors (TKI) [85]. The success indicated by these studies led to the recent approval of Nivolumab, which proved better in improving overall-survival and also was better tolerated when compared to everolimus in VEGF-refractory patients [86]. Moreover, the combination of Nivolumab with Ipilimumab for patients with intermediate or poor risk disease was also approved, again showing the value of combining blocking of several immune checkpoints [87,88]. Nowadays, one of the major challenges is proper selection of the patients that will experience the maximum benefit; this assessment depends on factors such as performance status of the patient and prognostic risk stratification, and there is no accurate (Level I evidence) biomarker for tailoring treatment in the clinic [89]. The value of PD-L1 immunoexpression for predicting response to therapy and as a prognostic marker is still a matter of debate [90]. Besides checkpoint inhibitors, clinical trials using vaccine-based therapies have been pursued, although clinical benefits are still to be reached [91].

### 3.4. Role of Immunoepigenetics?

Since many subtypes of RCC exist, it is also natural that their epigenetic background differs. For example, mutations in the methyltransferase SETD2 are typical of ccRCC, but not in the other RCC subtypes [92]. Indeed, inactivation of this histone-modifying enzyme results in increased tumor progression and aggressiveness, and poorer patient outcome [93]. To illustrate the different epigenetic background in the heterogeneity of RCC, a set of histone methyltransferases and demethylases is able to accurately discriminate among the various RCC subtypes and oncocytoma, an important differential diagnosis [94]. Also, several promoter-methylated genes involved in the various hallmarks of cancer are found in RCC, including the ones related to angiogenesis (in which VHL is included), metabolism, apoptosis and cell cycle, among others [95]. Agents targeting specific epigenetic aberrations have also shown anti-neoplastic activity in RCC, including the EZH2 inhibitor GSK126, which suppressed migration and invasion [96], and various HDAC inhibitors, alone or in combination with routinely used agents [97,98,99,100,101]. Moreover, similar to BlCa, these latter inhibitors also showed the ability to reverse resistance to currently used agents such as mTOR inhibitors [102]. 

A summary of most recent studies addressing combination strategies between epigenetics and immune environment in KCa is presented in Table 2 [103,104,105,106,107,108,109]. Since IL-2 has been used as a form of immunotherapy for KCa for more than one decade, several studies have explored its combination with epigenetic drugs, including methylation- and acetylation-targeting drugs. Indeed a phase I trial [103] disclosed that the combination of the demethylating agent decitabine and IL-2 was relatively safe (in patients with metastatic or unresectable melanoma and KCa). However, only five patients with KCa were enrolled, and three of them showed stable disease, which limits the clinical conclusions to be drawn from this trial. However, Kato et al. [105] explored another epigenetic mechanism in combination with IL-2; the use of a HDAC inhibitor (MS-275) was shown to result in a synergistic therapeutic effect in vitro and in an in vivo mouse model. Indeed, the combination led to an enrichment in CD4- and CD25-positive T cells and in decreasing of FoxP3-positive T regulatory cells (Tregs), and impeded the development of lung metastases in the mouse model, prolonging survival of the animal. Interestingly, the authors hypothesized about the synergism observed between these two agents based on opposite (but complementary) mechanisms: IL-2 enhances activation of effector T cells (which are reduced by MS-275), while MS-275 causes depletion of Tregs (which are potentiated by IL-2)—resulting in the end of a net antitumor effect mediated by increased effector cells and decreased Tregs. This has been, at least in part, confirmed for other HDAC inhibitors, such as entinostat, which downregulate FoxP3 and hence Tregs [108], leading to a phase 1/2 trial [107]. This study enrolled metastatic 47 ccRCC patients and showed a beneficial response with combining entinostat with IL-2 (objective response of 37%), showing the promises of such therapeutic approaches to metastatic ccRCC. Also, in this case, treatment reduced the number of Tregs, and lower amounts of these cells associated with better response to therapy.

In similarity to IL-2, IFN therapies (although less used nowadays) have also been shown to be improved when combined with epigenetic drugs. Decitabine was shown to overcome resistance to IFN treatment in melanoma and KCa cells, restoring apoptosis and increasing expression of IFN-related genes by ten times, including XAF1. Moreover, it increased the expression of CTAs, further enhancing the potential for combining with immune therapies [104]. Likewise, the HDAC inhibitor valproic acid also potentiated IFN signaling [106], showing that multiple epigenetic mechanisms can be used to enhance the same immune therapy approach.

More recently, Orillion A and Hashimoto A et al. [109] put in evidence that entinostat further potentiates the antitumor effect of immune checkpoint inhibitors, namely anti-PD-1, in both lung and renal cell carcinoma. The combination increased survival in vivo, shifting the immunosuppressive microenvironment into a tumor-suppressive one, specifically downregulating polymorphonuclear neutrophils and myeloid-derived suppressor cells. These results support the advantage of combining these epigenetic drugs with immune checkpoint inhibitors.

## 4. Prostate Cancer

### 4.1. Major Clinical Challenges

Prostate cancer (PCa) is not only the most common urological malignancy, but also the 2nd most incident (age standardized incidence rate of 29.3 per 100,000) and the 6th most deadly (age standardized mortality rate of 7.6 per 100,000) male cancer worldwide [110]. Furthermore, the biggest challenge is perhaps the one related to overtreatment: there is an urgent need for accurate predictive biomarkers of this disease, that may identify which patients will experience disease relapse, requiring more strict follow-up and adjuvant treatments [111]. This is a result, in great part, of the wide access to serum prostate-specific antigen (PSA) screening, responsible for detecting over 80% of PCa at localized stage, which displays excellent outcome [112]. 

Most of the time, when referring to PCa, one is referring to prostate acinar adenocarcinoma, by far the most common subtype ( >90% of the cases). Histological examination of prostatic biopsies and of the prostatectomy specimen is of paramount importance, because it dictates patients’ prognosis and the next interventions. Among these histological features, Gleason grading (already used for decades, with small modifications over time) [113] and, more recently, the prognostic grade groups proposed by the new World Health Organization classification, are good predictors of event-free survival [11,114,115]. Still, additional markers are needed to further discriminate subgroups of patients with a distinct outcome. 

On the other end of the spectrum, there is an additional challenge: dealing with metastatic, castration-resistant PCa (CRPC). In these patients, androgen receptor-related signalling is maintained despite the low levels of circulating androgens [116]. This is commonly accompanied by neuroendocrine differentiation of PCa and, more importantly, it is very hard to treat. Again, better biomarkers are needed for predicting which patients will transit into this phenotype (and when), and the molecular background of this disease must be better dissected, in order to find novel effective treatments, other than androgen deprivation therapy [117,118,119]. 

### 4.2. The Connection between Genetic, Epigenetic and Immune Landscape in Prostate Cancer

Overall, immune therapies have been less successful in PCa when compared to other urological malignancies; this has been attributed mainly to the overall “cold” tumor microenvironment in PCa, with low expression of immune checkpoint proteins, low tumor mutational burden and paucity of mutations in DNA repair genes. Also, the comonly observed deletion or mutation of PTEN contributes to the “immune desert” of PCa, since the latter activates IFN1-related pathways [120]. However, a recent large study of 9393 PCa samples indeed disclosed an immune-related tumor cluster, with distinct patient outcome [121]. In this study, the proportion of different immune cell populations was associated with distinct distant metastasis-free survival and, interestingly, PD-L2 emerged as a promising prognostic biomarker (contrarily to PD-L1, like in BlCa and RCC) and predictive factor (of response to radiotherapy), which may also mean it can be a promising therapeutic target [121]. Genomic aberrations in PCa do divide 74% of the disease into several groups, mostly based on presence of specific gene fusions (ERG, ETV1/4, and FLI1) and mutations (SPOP, FOXA1 and IDH1); however, PCa belongs overall to the low mutational burden class of tumors. The study pointed out, however, that among the 26% of the tumors without obvious molecular aberration there was evidence of DNA hypermethylation and mutations in epigenetic enzymes KDM6A and KMT2D [122]. Targeting the epigenetic landscape of PCa may help liberating this immune suppressive environment (see below), as illustrated by the influence of the methylatransferase EZH2, which inhibits T-lymphocyte chemoatractants (CXCL9 and CXCL10) and antigen presentation, thereby contributing to the “cold” microenvironment in CRPC with neuroendocrine differentiation [123].

### 4.3. Immune Therapies—Brief Overview in Prostate Cancer

Immune therapies have been explored in PCa for quite some time, including a variety of agents, such as cytokines (including IL-2) and viral and dendritic cell vaccines. In fact, the FDA has approved sipuleucel-T for treating minimally symptomatic metastatic CRPC already in 2010 [124]. Several of these strategies have employed prostate-specific (like PSA) or tumor specific antigens for directing responses [125]; for sipuleucel-T, which is an autologous cellular immunotherapy using the antigen presenting cell precursors from the patient, the target molecule is prostate acid phosphatase (PAP), and in vitro activated cells are reinfused in the patient, which resulted in a significant improvement in overall survival in the phase 3 IMPACT clinical trial [126]. Since then, several vaccine-based therapies have demonstrated promising results, and seem to especially benefit patients with a good prognostic disease and low disease burden (for extended review refer to [127]). With the advent of checkpoint inhibitors these agents were also tested in PCa [128]. These agents do not elicit a specific response against an individual target (contrarily to therapeutic vaccines) and, until now, they have not yet demonstrated convincing overall survival benefits in PCa patients. In a cohort of metastatic CRPC patients progressing under docetaxel the anti-CTLA-4 Ipilimumab was shown to produce an increase in overall survival, though, in a subgroup of patients with good prognostic features [129]. Novel strategies (including combination of agents, such as those targeting the PD-1/PD-L1 axis) are to be explored in the near future, and a multitude of clinical trials are underway [127]. 

### 4.4. Role of Immunoepigenetics?

The epigenetic background of PCa is very diverse [130]. Several gene promoters are consistently found to be hypermethylated in PCa, including for instance GSTP1, which can be detected in several bodily fluids, including plasma or urine [131,132,133,134]. Also, several microRNAs are involved in disease progression and aggressiveness [135,136]. From a therapeutic point of view, LSD1 (a lysine histone demethylase) has proved to be an interesting target, since its inhibitors were effective in preventing CRPC tumor growth in vitro and in vivo [137]. EZH2, which associates with poor prognostic features and worse patient outcome [112], is another target worth exploring. Moreover, both demethylating agents such as azacitidine, HDAC inhibitors such as vorinostat and even novel agents such as the pan-bromodomain inhibitor JQ1 have shown antitumor effect in prostate cancer models, including CRPC [138,139,140].

A summary of most recent studies addressing combination strategies between epigenetics and immune environment in PCa is depicted in Table 3 [108,141,142,143,144,145,146,147,148,149,150,151,152]. Some studies have focused on epigenetic regulation of response to IFN, where both methylation and acetylation can be involved, as demonstrated by Dunn and collaborators [141]. The authors showed that treatment with both decitabine and trichostatin (TSA) restored JAK1 levels, which proved to be necessary for IFN cascade to occur. Danziger et al. [147] further elaborated on this; they showed that the same epigenetic modifiers partially restored IFN signaling but, very relevantly, they also showed that this attenuated, but did not completely block viral infection in PCa cells. Given the known antitumor effect, the authors then conclude that these epigenetic drugs may be considered for combination with therapeutic viruses, since infection of cells is still possible. Moreover, because therapeutic vaccines have shown promising results for PCa treatment, the work of Shen and collaborators [108] is valuable, since combination of the HDAC inhibitor entinostat with the SurVaxM peptide vaccine led to increased beneficial effects in a CRPC model, both by increasing effector cells (CD8-positive cells) and IFN signaling, but also by inhibiting Tregs.

Like in other tumor models mentioned above, decitabine was also demonstrated to induce the expression of CTAs in PCa, in a synergistic combination with the HDAC inhibitor panobinostat [149]. In this study, the authors created an ex vivo culture system of PCa biopsies, which further showed inducing of CTAs, namely SSX2, in all nine cultured specimens (originating from 9 different patients). Moreover, circulating tumor cells of two out of eleven patients with PCa disclosed the presence of SSX2 mRNA. Epigenetic drugs might then result in making PCa cells more immunogenic and amenable to targeting by immune therapies. Similar results were obtained by Sulek et al. [152], which additionally demonstrated that combining lenalidomide with decitabine induced dendritic cells activity and ability to stimulate T cells. 

Chemokines that attract effector and regulatory cells to the tumor are very relevant in regulating immune response. Like for CTAs, decitabine also restored/induced the expression of the chemokine CXCL14, which functions as a chemoattractant for dendritic cells [145]. On the other hand, Su and coworkers [151] have focused on CCL2. In their work, they uncovered that the polycomb repressor complex 1 (PRC1) in androgen-receptor and neuroendocrine double-negative PCa is responsible for producing metastases, by regulating CCL2 expression. CCL2 is also involved in the recruitment of immune cells, this time of M2-like tumor-associated macrophages and Tregs, which are immunosuppressive. Elegantly, the authors showed that a PRC1 inhibitor (GW-516) combined with double-inhibition of PD-1 and CTLA-4 led to metastases suppression in vivo.

Recently, among the epigenetic modifiers, those targeting bromodomains have been gathering some attention, including in PCa [153]. Mao et al. has recently demonstrated that JQ1, an inhibitor of bromodomain and extra-terminal (BET) bromodomain family, impacts on the immune response players, including PD-L1 downregulation, MHC1 upregulation, additive effect to anti-CTLA-4 agents and inducing an increase in the CD8/Treg ratio, leading to immunogenicity [142].

The development of animal models allowing for the study of the relation between PCa and inflammation have proved very useful for better understanding the underlying mechanisms and for reaching relevant biomarkers to be validated in human patient samples [154]. For instance, Zhao et al. were able to study and propose a regulatory axis of inflammation-associated PCa, based on a Twist1-dependent DNMT3a recruitment to the promoter of miR-186, inducing its methylation and blocking the response of this microRNA to inflammation (inhibiting the transcription of NF-kB/p65) [155].

Besides more conventional drugs, natural compounds have been shown to display antitumor properties [156,157,158], namely concerning epigenetic regulation. As an example, the natural methanolic extract from P. foetida leaves was shown to have antitumor effects in PCa cell lines, mechanistically resulting in downregulation of DNMT1, HDACs and various pro-inflammatory cytokines, including IL-6 and TNF-α. Authors believe that these compounds may, therefore, target epigenetic landscape of tumors, but also be used for promoting immune responses [159].

## 5. Testicular Cancer

### 5.1. Major Clinical Challenges

When refering to testicular cancer one is often referring, in fact, to testicular germ cell tumors (TGCTs), since they represent more than 95% of all testicular neoplasms. Despite not being a common malignancy, they are the most frequent solid cancers among young adult Caucasian men and incidence is increasing worldwide due to changes in lifestyle, which is in line with the proposed genetic and environmental model (“genvironment”) of the disease [160,161,162]. 

TGCTs are very heterogeneous [163], reflecting a complex tumor model that closely resembles developmental biology phenomena. Type II TGCTs (the most common) derive from a precursor lesion named germ cell neoplasia in situ (GCNIS), which then evolves into two big categories, seminomas (SEs) and non-seminomas (NSs), the latter being further divided into embryonal carcinoma (EC), postpubertal-type yolk sac tumor (YST), choriocarcinoma (CH) and postpubertal-type teratoma (TE) [162,164]. An integrated approach to TGCTs [165] has evidenced remarkable differences in methylation profiles between SE and NS, and also confirmed the value of the miR371–373 cluster in these tumors. One of the major challenges of TGCTs is common to other pediatric malignancies: they do not show frequent mutations, and copy number variations are not abundant. This way, the quest for finding disease biomarkers (for diagnostic, prognostic or even therapeutic purposes) can only be acomplished by turning into epigenetics (since the epigenetic background of these tumors is very rich) [166].

The overall prognosis of TGCTs is excellent, with survival rates frequently over 90%. However, many challenges lie ahead; overtreatment of indolent disease that would be cured by orchiectomy alone is a major problem, since these cancers afflict mainly young patients with high chances of becoming cancer survivors and experiencing late side effects of systemic and radiation treatments [167]. So, there is an urgent need for better biomarkers to accurately follow-up stage I disease and to discriminate the subgroup of patients that might experience relapse, and that can benefit from adjuvant treatment. Furthermore, a subgroup of patients develops cisplatin-resistant disease, which is very difficult to handle and treat, being responsible for most of the deaths. More effort needs to be put into understanding the biology of this disease phenotype and into findings novel treatment options for these patients [168].

### 5.2. The Immune Landscape and Immune Therapies—Brief Overview in Testicular Germ Cell Tumors

TGCTs, namely SEs, are characterized by an often remarkable immune infiltrate; however, immune checkpoint expression and immune therapies are still scarcely explored in these tumors. Few studies have shown PD-L1 (but not PD-1) expression in TGCTs, both in immune cells and tumor cells. Importantly, this has been shown to associate with prognosis, namely survival outcome of the patients, which means that there seems to be a rationale for using immune therapies, alone or in combination, to treat these patients [169,170,171]. Also, CTLA-4 was shown to be expressed in these neoplasms [172].

In this line, clinical trials were put in motion to assess the efficacy of immune checkpoint inhibitors for treating TGCT patients; these trials were, however, unsuccessful, as there was no improvement in survival [173,174,175]. However, they have been pursued in unselected populations of multiple relapsed cisplatin-resistant patients. Also, there are preliminary results indicative of some clinical activity for the combination of durvalumab and tremelimumab [174]. Further combinations should be explored in the future to assess the value of these agents in treating this disease.

### 5.3. Role of Immunoepigenetics?

The epigenetic landscape of TGCTs is very determinant in these tumors, from their genesis to progression, recapitulating developmental events. DNA and histone modifications in these tumors are quite distinct, SEs being hypomethylated and more acetylated, while NSs show hypermethylation and increased acetylation [176,177]. Given their supranumerical X-chromosome content, the expression of XIST, triggered by demethylation of its promoter, is maintained in these tumors, contrarily to somatic cancers [178], which may be used as a liquid biopsy marker of the disease [179]. In this field, a lot of attention has been directed to microRNAs, since they compensate for the limitations of the classical serum markers in diagnostic, prognostic and follow-up settings [180]. Some studies on agents targeting histone (de)acetylation were reported, showing that trichostatin A led to increased apoptosis of EC cells in synergy with retinoic acid [181]; moreover, the SE-like cell line TCam-2 proved to be resistant to apoptosis and differentiation when exposed to decitabine, a demethylating agent, but sensitive to the HDAC-inhibitor depsipeptide [182].

For the time being, no data on combination of immune therapies, such as checkpoint inhibitors, and epigenetic drugs is available; this means that there is a lot to explore in this field, since there is rationale (and need) for novel therapies, particularly to mitigate cisplatin resistance. Indeed, the ability of demethylating agents (like decitabine) to induce immunogenicity by restoring expression of CTAs (like mentioned above for the other tumor models) might indeed be useful for applying to NSs, since these subtypes show increasingly more hypermethylation in a differentiation-dependent manner, contrarily to SEs which are highly hypomethylated [183]. This is in line with the absence of expression of CTAs in all NSs in the study by Bode et al., as opposed to their expression in a subset of SEs (ranging from 3% to 40% depending on the specific CTA) [184]. Moreover, given the effects on Tregs and effector T cells produced by agents like entinostat in the other tumor models, and since TGCTs often show a rich immune infiltrate, it is fair to assume that combination of such agents with immune therapies might result in a more robust combination.

## 6. Conclusions: Future Perspectives for Immunoepigenetics in Urological Cancer

**Figure 1:**
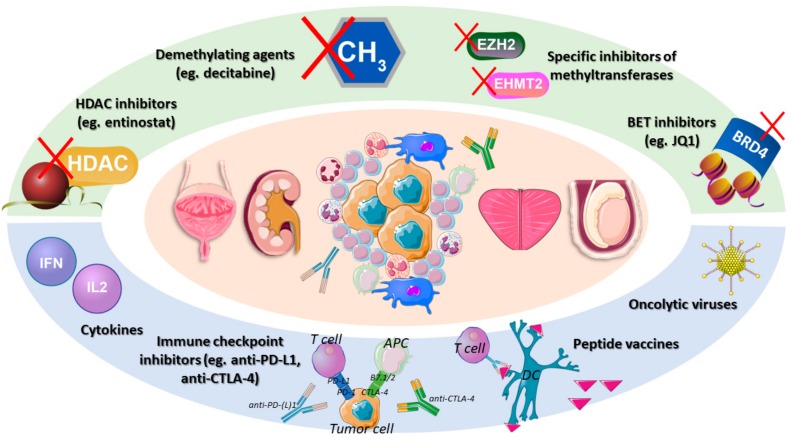
Immunoepigenetic therapeutic strategies for urological malignancies. Since there is evidence that the tumor immune response is epigenetically regulated, there is rationale for combining epigenetic modifiers with immune therapies, in order to achieve better clinical outcome. Abbreviations: APC—antigen presenting cell; BET—bromodomain extra-terminal; CTLA-4—cytotoxic T-lymphocyte associated antigen; DC—dendritic cell; HDAC—histone deacetylase; PD-(L)1—programmed death (ligand) 1.

We have reviewed recent literature that provided evidence for interactions between epigenetic regulation and the immune microenvironment, and that explored the combination of immune therapies with epigenetic therapies, showing promising (synergistic) results in vitro and in vivo. Several strategies were presented (Figure 1) for BlCa, KCa, and PCa, holding the promise of combining various immune therapies, from cytokines, to immune checkpoints, to therapeutic vaccines and oncolytic viruses, with the currently available epigenetic modifiers, mainly the well-known demethylating and HDAC inhibitor agents, but also other specific agents targeting bromodomains or specific players (EZH2, for instance). For TGCTs, the same combinations are expected to be attempted in the future, especially if immune therapies consistently prove their value in future works. For these tumors, epigenetic drugs are already being actively explored owing to their ability to modulate the rich and characteristic epigenetic landscape of each tumor entity.

The knowledge that the immune response is epigenetically regulated should lead to efforts of therapeutically targeting both these cancer hallmarks in combination, to achieve a better clinical outcome, and the choice of the best therapy should reflect the molecular subtype and genomic background of each tumor [82,185]. Several clinical trials are ongoing in various tumor models, and will shed light on this subject, including for urological malignancies [186,187,188].

## Figures and Tables

**Table 1:** Immunoepigenetic-based studies in bladder cancer.

Epigenetic Target/mechanism	Immune Therapy/Target	Major Finding	Methods	Sample Type and Size	Author
Methylation (decitabine) and acetylation (TSA)	9 CTAs	The most expressed antigens are the MAGE-A families Expression of CTAs is induced by decitabine	RT-PCR WB	Cell lines BlCa and normal urothelium tissues (*n* = 56)	Fradet Y, 2006 [54]
Methylation (25 tumor-suppressor genes)	Response to BCG-therapy	Methylation status of several targets predicted response to BCG-therapy and disease recurrence in T1 G3 NMIBC	MS-MLPA	91 BlCa tissues	Agundez M, 2011 [55]
PMF-1 methylation	Response to BCG-therapy	Unmethylated PMF-1 associated with failure of BCG therapy (disease recurrence in T1 high-risk NMIBC)	qMSP	108 BlCa tissues	Alvarez-Múgica M, 2013 [56]
Methylation (57 targets)	Response to BCG-therapy	Methylation status of several targets predicted response to BCG-therapy and disease recurrence in high-grade NMIBC	MS-MLPA	82 BlCa and 13 normal urothelium tissues	Husek P, 2017 [57]
DNA methylation-derived index	Neutrophil-to-lymphocyte ratio	Higher methylation index associated with disease outcome in BlCa	Bioinformatics	DNA methylation data from leukocyte subtypes	Koestler DC, 2017 [61]
Methylation (decitabine)	IL-6	Decitabine leads to NOTCH1 demethylation and expression, leading to IL-6 release	WB RT-qPCR Methylation array RNA-seq Pyrosequencing ELISA FACS-sorting	Tissues (BlCa + normal urothelium, *n* = 174) + in vitro (cell lines)	Ramakrishnan S, 2019 [64]
FOXP3, IFNG, IL13, IL17A (methylation)	CD4+ T cells in BlCa	↑ CD4+ T cell lineage commitment assessed by CpG methylation associates with better prognosis Hypomethylation of the four targets in CD4+ T cells associated with complete response to CT	FACS-sorting Pyrosequencing 5mC ELISA	BlCa tissues (*n* = 22), LNs (*n* = 76) and blood (*n* = 48)	Bergman EA, 2018 [58]
PRF1 methylation	Tissue-resident memory CD8+ T cells in BlCa	These cells are epigenetically cytotoxic and show signs of exhaustion (show ↓ methylation levels of PRF1 and ↑ PD-L1 expression)	FACS-sorting Pyrosequencing	BlCa tissues, LNs and blood (*n* = 53 patients)	Hartana CA, 2018 [59]
Knockout of lncRNA UCA1	Knockout of PD-1	Combined UCA1 and PD-1 knockout resulted in synergistic antitumor effect by favoring an immunostimulatory microenvironment	CRISPR-Cas9 FACS-sorting RT-PCR WB	In vitro (cell lines) + in vivo (mouse)	Zhen S, 2018 [60]
EZH2 inhibition (EPZ011989)	NK cells	BlCa cells with KDM6A and SWI/SNF mutations are frequent and show overactivation of EZH2 EZH2 inhibition in these cells resulted in ↑ immune signature (IFN-γ) and activated NK signaling, resulting in MIBC cell death	WB RNA-sequencing IHC	In vitro (cell lines) + in vivo + BlCa tissues	Ramakrishnan S, 2019 [62]
EHMT2/DNMT inhibition (CM-272, A-366, decitabine)	Anti-PD-L1	Dual targeting of EHMT2/DNMT leads to immunogenic cell death (conversion into a “hot tumor”), and this is potentiated by combining with anti-PD-L1 ↑ EHMT2 expression leads to resistance to anti-PD-L1	RT-qPCR WB/Dot blot ChIP IF/IHC FACS-sorting ELISA Whole transcriptome analyses Pyrosequencing	In vitro (cell lines) + in vivo (mouse) + BlCa tissues (*n* = 87 patients)	Segovia C, 2019 [63]

**Abbreviations**: 5mC—5-methylcytosine; BCG - Bacillus Calmette-Guérin; BlCa—bladder cancer; ChIP—chromatin immunoprecipitation; CT—chemotherapy; CTA—cancer testis antigen; ELISA -enzyme-linked immunosorbent assay; FACS—fluorescence-activated cell sorting; IF—immunofluorescence; IFN-γ—interferon gamma; IHC—immunohistochemistry; IL-6—interleukin 6; LN—lymph node; lnCRNA—long non-coding RNA; MIBC—muscle-invasive bladder cancer; MS-MLPA—methylation-sensitive multiplex ligation-dependent probe amplification; NMIBC—non muscle-invasive bladder cancer; PD-1—programmed cell death protein 1; PD-L1—Programmed death-ligand 1; qMSP—quantitative methylation-specific PCR; RT-(q)PCR—real-time quantitative polymerase chain reaction; TSA—trichostatin A; WB—Western Blot.

**Table 2:** Immunoepigenetic-based studies in Kidney Cancer.

Epigenetic Target/Mechanism	Immune Therapy/Target	Major Finding	Methods	Sample Type and Size	Author
Methylation (decitabine)	IL-2	Phase I trial: safe combination; decitabine may increase activity of IL-2	Pyrosequencing DNA microarray	Blood (*n* = 5 patients)	Gollob JA, 2006 [103]
Methylation (decitabine)	IFNs	Synergistic effect, overcoming resistance to IFN-based therapy (same effect with antisense to DNMT1) Reactivation of CTAs after DNMT1 depletion	WB RT-PCR MSP	In vitro (cell lines) + in vivo (mouse)	Reu FJ, 2006 [104]
Acetylation (HDAC inhibitor MS-275)	IL-2	Synergistic antitumor effect	FACS-sorting	In vitro (cell lines) + in vivo (mouse)	Kato Y, 2007 [105]
Acetylation (VA)	IFN-alpha	Combination with VA altered gene expression (↑ expression chemokines)	Gene expression array RT-qPCR	In vitro (cell lines)	Juengel E, 2011 [106]
Acetylation (entinostat)	IL-2	Entinostat ↓ Foxp3 levels in Tregs, enhancing antitumor effect of IL-2 (STAT3 signaling involved)	FACS-sorting WB IP IHC RT-qPCR	In vitro (cell lines) + in vivo (mouse)	Shen L, 2012 [108]
Acetylation (entinostat)	PD-1 (inhibitor)	Entinostat enhances the antitumor effect of anti-PD-1 therapy (↓ immunosuppressive cell populations - MDSCs)	FACS-sorting WB IP RT-qPCR	In vitro (cell lines) + in vivo (mouse)	Orillion A and Hashimoto A, 2017 [109]
Acetylation (entinostat)	IL-2	Phase 1/2 trial: objective beneficial response with the combination in patients with metastatic ccRCC Entinostat ↓ the number of Tregs and ↑ APCs, associating with response	FACS-sorting IHC	Blood (*n* = 47 patients) + ccRCC tissues	Pili R, 2017 [107]

**Abbreviations**: ccRCC—clear cell renal cell carcinoma; DNMT1—DNA methyltransferase 1; FACS—fluorescence-activated cell sorting; HDAC—histone deacetylase; IFN—interferon; IHC—immunohistochemistry; IL-2—interleukin-2; IP—immunoprecipitation; MDSC—myeloid-derived suppressor cells; MSP—methylation-specific PCR; PD-1—programmed cell death protein 1; PD-L1—Programmed death-ligand 1; RT-(q)PCR—real-time (quantitative) polymerase chain reaction; Tregs—regulatory T cells; VA—valproic acid; WB—Western Blot.

**Table 3:** Immunoepigenetic-based studies in Prostate Cancer.

Epigenetic Target/Mechanism	Immune Therapy/Target	Major Finding	Methods	Sample Type and Size	Author
Methylation and acetylation (silencing of JAK1 kinase)	IFN	Treatment with decitabine and TSA induced JAK1 expression, making cells responsive to IFN therapy	WB IP Northern blot IF	In vitro (cell lines)	Dunn GP, 2005 [141]
Methylation (decitabine)	CXCL14	Decitabine restored CXCL14 expression and function (chemoattractant to DC)	Affinity chromatography IHC/ICC RT-qPCR MSP Bisulfite sequencing	Tissues (total: *n* = 24) + in vitro (cell lines)	Song EY, 2010 [145]
Acetylation (VA)	IFN-alpha	Combination with IFN-alpha enhances the antitumor effect of VA (growth, adhesion, migration)	RT-qPCR WB FACS-sorting	In vitro (cell lines) + in vivo (mouse)	Hudak L, 2012 [143]
Acetylation (vorinostat, entinostat)	T-cell mediated lysis	Exposure to vorinostat or entinostat enhances T-cell mediated death	WB FACS-sorting	In vitro (cell lines)	Gameiro SR, 2016 [146]
Methylation	CXCL12	Methylation of CXCL12 promoter associates with poor outcome in PCa, including BCR-free survival	qMSP IHC	PCa tissues (*n* = 247 patients) + TCGA cohort (*n* = 498 patients)	Goltz D and Holmes EE, 2016 [148]
Methylation (decitabine) and acetylation (TSA)	IFN (through JAK1 kinase)	IFN signaling is epigenetically regulated in PCa Epigenetic modifiers partially restore IFN-sensitivity and attenuate (but not completely block) viral infection	RT-qPCR WB IF FACS-sorting DNA sequencing	In vitro (cell lines)	Danziger O, 2016 [147]
Methylation (decitabine) and acetylation (panobinostat)	CTAs	Treatment induces expression of CTAs (synergistically) Expression of the CTA SSX2 in CTCs from PCa patients	RT-qPCR Bisulfite-sequencing FACS-sorting	In vitro (cell lines and ex vivo PCa culture) + blood (*n* = 11 patients)	Heninger E, 2016 [149]
Methylation	DEFB1 (mediator of innate immunity)	Epigenetic regulation of DEFB1 by promoter methylation	Bisulfite-sequencing Pyrosequencing RT-qPCR IHC WB	Tissues (*n* = 60 patients) + in vitro (cell lines)	Lee J, 2016 [150]
Methylation (5-AZA)	CTAs, DCs	Treatment induces expression of CTAs Combination with lenalidomide induces DC function	Gene microarray RT-qPCR FACS-sorting ELISA WB	In vitro (cell lines) + in vivo (mouse)	Sulek JE, 2016 [152]
Acetylation (entinostat)	Peptide vaccine (SurVaxM)	Entinostat ↓ Foxp3 levels in Tregs, enhancing antitumor effect of SurVaxM in a CRPC model ↑ antigen-specific CD8 cells + ↑ IFN immune response	FACS-sorting WB IP IHC RT-qPCR	In vitro (cell lines) + in vivo (mouse)	Shen L, 2017 [108]
Methylation (silencing of SERPINB1, the endogenous inhibitor of NE)	NE	DNA and histone methylation (DNMT- and EZH2-mediated) silence SERPINB1 in PCa, contributing to inflammation-induced PCa progression	WB RT-qPCR ChIP Pyrosequencing FACS-sorting IHC/IF	In vitro (cell lines) + in vivo (mouse)	Lerman I, 2019 [144]
PRC1 (inhibitor GW-516)	CCL2; PD-1 and CTLA-4 double inhibitor	PRC1 drives metastases by inducing CCL2, which in turn enhances recruitment of immunosuppressive M2-like TAM and Tregs Combined inhibition of PRC1 and checkpoint inhibitors suppresses metastases	IHC IF RNA-seq ChIP-seq FACS-sorting IB/WB RT-qPCR	PCa tissues + in vitro (cell lines) + in vivo (mouse)	Su W, 2019 [151]
BET bromodomain inhibitor (JQ1)	PD-L1, HLA-ABC, CTLA-4	↓ PD-L1 expression, ↑ MHC 1 Effect additive to anti-CTLA-4 treatment ↑ CD8/Treg ratio	RNA-seq RT-qPCR FACS-sorting	In vitro (cell lines) o in vivo (mouse)	Mao W, 2019 [142]

**Abbreviations**: 5AZA—5-azacytidine; BCR—biochemical recurrence; BET—bromodomain and extra-terminal motif; ChIP—chromatin immunoprecipitation; ChIP-seq—ChIP sequencing; CRPC—castration-resistant prostate cancer; CTA—cancer testis antigen; CTC—circulating tumor cell; CTLA-4—cytotoxic T-lymphocyte associated antigen; DC—dendritic cells; ELISA - enzyme-linked immunosorbent assay; FACS—fluorescence-activated cell sorting; IB—immunoblot; ICC—immunocytochemistry; IF—immunofluorescence; IFN—interferon; IHC—immunohistochemistry; NE—neutrophil elastase; PD-1—programmed cell death protein 1; PCa—prostate cancer; PD-L1—Programmed death-ligand 1; PRC1—polycomb repressor complex 1; qMSP—quantitative methylation-specific PCR; RNA-seq—RNA-sequencing; RT-qPCR—real-time quantitative polymerase chain reaction; TAM—tumor associated macrophage; Treg—regulatory T cells; TSA—trichostatin A; VA—valproic acid; WB—Western Blot.

## Abbreviations

BlCa	Bladder cancer
NMIBC	Non muscle invasive bladder cancer
MIBC	Muscle invasive bladder cancer
BCG	Bacillus-Calmette Guérin
FDA	Food and Drug Administration
PD-1	Programmed death 1
PD-L1	Programmed death-ligand 1
CTLA-4	Cytotoxic T lymphocyte-associated antigen
APC	Antigen-presenting cells
EMT	Epithelial-to-mesenchymal transition
HDAC	Histone deacetylase
CTA	Cancer testis antigen
IFN	Interferon
DC	Dendritic cell
DNMT	DNA methyltransferase
KCa	Kidney cancer
RCC	Renal cell carcinoma
ccRCC	Clear cell renal cell carcinoma
pRCC	Papillary renal cell carcinoma
chRCC	Chromophobe renal cell carcinoma
IL	Interleukin
TKI	Tyrosine kinase inhibitor
Treg	T regulatory cell
PCa	Prostate cancer
PSA	Prostate-specific antigen
CRPC	Castration-resistant prostate cancer
TSA	Trichostatin
BET	Bromodomain Extra-Terminal
TGCT	Testicular germ cell tumor
SE	Seminoma
NS	Non-seminoma
EC	Embryonal carcinoma
YST	Postpubertal-type yolk sac tumor
CH	Choriocarcinoma
TE	Postpubertal-type teratoma
